# Bullying among nursing professionals in Brazil: validity and reliability of the Negative Acts Questionnaire-Revised

**DOI:** 10.47626/1679-4435-2023-1219

**Published:** 2024-02-16

**Authors:** Roberta Nazario Aoki, Dirceu da Silva, Edinêis de Brito Guirardello

**Affiliations:** 1 Faculdade de Enfermagem, Universidade Estadual de Campinas (UNICAMP), Campinas, SP, Brazil; 2 Faculdade de Educação, UNICAMP, Campinas, SP, Brazil

**Keywords:** nursing, bullying, validation study, psychometrics, factor analysis, statistical, enfermagem, bullying, estudos de validação, psicometria, análise fatorial

## Abstract

**Introduction:**

Bullying in the nursing work environment has negative consequences for both professionals and institutions. The early identification of this behavior can contribute to a positive organizational climate and better quality of life.

**Objectives:**

This study analyzed the validity and reliability of the Negative Acts Questionnaire-Revised with nursing professionals.

**Methods:**

A total of 350 nursing professionals were included in this methodological study. Multivariate confirmatory factor analysis was based on 4 domains, as in the Portuguese version of the Negative Acts Questionnaire-Revised. The instrument consists of 22 items that address negative acts committed in the work environment without directly mentioning bullying. Respondents indicate, on a Likert-type scale, how often they experience these acts in their work routine.

**Results:**

The adjusted model of the Brazilian version of the Negative Acts Questionnaire-Revised resulted in an instrument with 20 items and 4 distinct domains that presented satisfactory validity and reliability for identifying bullying behavior among nursing professionals.

**Conclusions:**

The Brazilian version of the Negative Acts Questionnaire-Revised is a valid instrument for identifying acts of bullying among nursing professionals and can be used in efforts to prevent such behavior in health services.

## Abstract (pt)

**Introdução:**

*bullying* no ambiente de trabalho da enfermagem acarreta consequências negativas para o profissional e para a instituição. A identificação precoce desse comportamento pode contribuir com um clima organizacional positivo e a qualidade de vida do profissional.

**Objetivos:**

Analisar a validade e confiabilidade do Negative Acts Questionnaire-Revised com profissionais de enfermagem.

**Métodos:**

Estudo metodológico com 350 profissionais de enfermagem. Para a análise, foi utilizada a técnica multivariada de análise fatorial confirmatória a partir de quatro dimensões, de acordo com a versão portuguesa do Negative Acts Questionnaire-Revised. O instrumento é composto por 22 itens que abordam atos negativos praticados no ambiente de trabalho sem menção direta ao *bullying.* Os profissionais são convidados a assinalar, em uma escala tipo Likert, com qual frequência vivenciam esses atos em sua rotina de trabalho. Resultados: O modelo ajustado do Negative Acts Questionnaire-Revised - versão brasileira resultou em instrumento composto de 20 itens e com quatro domínios distintos, que apresentaram validade e confiabilidade satisfatórias para a identificação de comportamentos de *bullying* em profissionais de enfermagem.

**Conclusões:**

O Negative Acts Questionnaire-Revised - versão brasileira é um instrumento válido para a identificação de atos de *bullying* entre profissionais de enfermagem e pode tornar-se uma ferramenta para a prevenção desse comportamento nos serviços de saúde.

## INTRODUCTION

Workplace bullying is characterized by systematic intimidating behavior by subordinates, colleagues, or superiors that can cause persistent and serious social, psychological, and psychosomatic problems to the targets of these acts.^1^ In addition to hurting the victims, the presence of bullying also damages organizational effectiveness, as is associated with absenteeism, work disengagement, increased turnover, and decreased productivity over time.^2^

The average prevalence of bullying at work is estimated at 14.6% worldwide, varying from 11.3 to 18.1% depending on the approach used to identify acts of intimidation in professional environments.^3^ The prevalence of bullying has been estimated at 61.9%^4^ in the health sector and varies considerably among nursing professionals (2.4 and 81%), depending on the region.^5^

In a study of 438 Russian nurses, 63% reported having suffered bullying at some point in their careers.^6^ An American study found that 40% of nurses also identified themselves as bullying victims,^7^ indicating a high rate of such behavior in health services. Reports of workplace bullying among nurses have been associated with lower quality of care and lower patient satisfaction and safety.^8^

Bullying among nursing professionals has negative consequences for both mental and physical health, including anxiety, depression, stress, insomnia, gastrointestinal problems, headaches, and hypertension.^2,9^ Bullying can also lead to a loss of confidence and self-esteem and high turnover rates.^10^ A lack of interaction among the work team and disengagement from nursing care routines can lead team members to feel unable to face challenges and can lead to errors in patient care, as well as to quitting their current job or the profession altogether.^10^

Due to the negative consequences of bullying for nursing professionals and patients, health service managers must seek effective strategies to contain and prevent these behaviors in the work environment. Thus, organizational interventions that focus on social support, educating health service leaders and managers, and creating and maintaining a positive work environment for nurses should be encouraged.^11,12^

The Negative Acts Questionnaire-Revised (NAQ-R), which was designed to identify the occurrence of workplace bullying, stands out among instruments to assess bullying.^13^ The original version of the NAQ-R, which included 22 items and was initially presented in a single-domain model,^14^ was subsequently expanded into 3 domains: personal bullying, work-related bullying, and physical forms of bullying.^13^ This instrument has been validated in different cultures and varies in the number of domains, for example, in some European and Asian countries it consists of 3 domains,^15,16^ while 4 are used in Portugal.^17^ However, all formats could identify bullying in their respective populations.

The NAQ-R has been widely used in international research to assess bullying among coworkers, especially health care professionals, who have been recognized as targets of bullying in several countries.^12,18,19^ In Brazil, few studies have used specific measures to assess bullying among nursing professionals. Although a single-domain version of the NAQ-R has been validated for Brazil,^20^ it has not been validated with health professionals. Therefore, this study aims to evaluate the measurement properties of the NAQ-R among Brazilian nursing professionals.

## METHODS

This methodological study was conducted at an educational institution linked exclusively with the Brazilian Unified Health System. Due to the instrument's 22 variables (items), a minimum sample of 110 subjects was recommended (ie, 5 times the number of variables). However, a total of 350 participants were included to ensure a satisfactory data set.^21^

Professionals aged 18 years or over who had been working at the institution for ≥6 months were considered eligible to participate in the study; those who were on leave or vacation were excluded.

The Brazilian NAQ, with 22 items that describe certain negative behaviors in the workplace,^13^ was used for data collection.^20^ Participants respond about their experience during the last 6 months of work in the unit, using a Likert scale with the following options: never (l point), once in a while (2 points), monthly (3 points), weekly (4 points), or daily (5 points).^13^ In the previous Brazilian validation study, the internal consistency was 0.90 according to Cronbach's alpha.^20^

Data were collected between April and June 2018. Professionals who met the inclusion criteria were invited to participate in the study and received an envelope containing the consent form, a form with personal and professional data, and the NAQ-R instrument, which were returned in sealed envelopes to one of the researchers, who coded the participants for data transfer to an electronic database.

The collected data were coded, categorized, and entered into an Excel spreadsheet (Microsoft, Redmond, WA, USA) and were subsequently exported and analyzed using IBM SPSS Statistics 22.0 (IBM, Armonk, NY, USA). Descriptive analysis involved calculating the absolute frequency and percentage values for categorical variables and position measurements (mean, maximum, and minimum) and dispersion measurements (SD) for continuous variables.

The 4-domain model used in the Portuguese NAQ-R was used to evaluate the measurement properties of the Brazilian version: Exclusion (8 items), Harassment (8 items), Quality/Overload (3 items), and Undervaluation (2 items).^17^ This decision was due to similarities in study population (nurses) and language (Portuguese) between Brazil and Portugal.

The structural validity of the NAQ-R was assessed through 2-stage confirmatory factor analysis: convergent and discriminant validity, considering the instrument's 4 domains. Structural equation models were based on the partial least squares estimation method using Smart PLS 3.2.1 (SmartPLS GmbH, Oststeinbek, Germany).^22^

To evaluate the convergent validity of the NAQ-R items, the results of the average variance extracted (AVE) were examined. Values > 0.50 indicate that the model is progressing towards a satisfactory result. The factor loadings between the items and their respective factors were then analyzed. Items with loadings < 0.50 were excluded.^23^

Discriminant validity was assessed using the Fornell & Larcker criterion, ie that the square roots of the AVEs are greater than the correlations between the factors.^24^ Cross-loadings were also analyzed to determine whether the factor loading of a specific item was higher in the factor to which it was initially assigned than in the other factors in the model.

After calculating the Pearson coefficient to determine the variation of the dependent variables, the following were analyzed: predictive validity (Q2), which measures the model's precision, with values greater than 0 indicating predictive relevance; and effect size (f² or Cohen's indicator), which assesses the importance of each construct in adjusting the model; values of 0.02, 0.15, and 0.35 were considered small, medium and large, respectively.^22^ In the final stage of the structural model, the path coefficients were interpreted to reveal the predictive relationship between the independent and dependent variables.

The study was approved by the institutional research ethics committee (decision 2,549,239). All participants provided written informed consent, as recommended in National Health Council Resolution 466/2012.

## RESULTS

A total of 350 nursing professionals participated in the study: 118 (34%) nurses and 232 (66%) licensed practical nurses. Their mean age was 40.2 years (SD = 8.95), 295 (84.3%) were women and 55 (16.7%) were men. Regarding the length of experience at the institution, 273 (78%) reported >4 years.

The first round of the model indicated that the AVE values (0.535-0.584) met the convergent validity criterion (AVE ≥ 0.500).^24^ Other model quality values, including the composite reliability and Cronbach's alpha, also proved adequate (Table 1 - Initial model).

**Table 1.** Convergent validity of the factorial model of the Brazilian Negative Acts Questionnaire-Revised (NAQ-R), Campinas, SP, Brazil

Domains	Initial model			Final model		
	AVE	Composite reliability	Cronbach's alpha	AVE	Composite reliability	Cronbach's alpha
Exclusion	0.535	0.900	0.871	0.540	0.890	0.853
Harassment	0.575	0.904	0.876	0.602	0.901	0.867
Quality/Overload	0.542	0.825	0.718	0.542	0.825	0.718
Undervaluation	0.584	0.808	0.648	0.584	0.808	0.648

AVE = average variance extracted.

However, when proceeding to the next stage, the discriminant validity assessment, 2 domains did not meet the Fornell & Larcker criterion^24^ (ie, that the square roots of the AVE of each dimension must be higher than their correlations with the others). Thus, to obtain discriminant validity, the variables NAQ7 and NAQ10 were removed from the Harassment and Exclusion domains, respectively, following the recommendations of Hair et al.^23^ Hence, the values in Table 1 changed slightly for these domains, but remained adequate (Table 1 - Final model). The discriminant validity values are presented in Table 2.

**Table 2.** Discriminant validity according to the criteria of Fornell & Larcker,^24^ Campinas, SP, Brasil

Domains	Exclusion	Intimidation	Quality/Work overload	Undervaluation
Exclusion	**0.735**			
Harassment	0.708	**0.776**		
Quality/Overload	0.702	0.668	**0.736**	
Undervaluation	0.608	0.612	0.675	**0.764**

Bold values are the square roots of the average variance extracted.

In the next stage, after the discriminant validity had been confirmed, the model and values were analyzed by calculating Pearson's coefficient (R²), Q2, and f². The significance level was set at 5% for all statistical tests. The results of this stage are presented in Table 3.

**Table 3.** Pearson's coefficient (R²) and indicators of predictive validity (Q2) and effect size (f²) in modeling of the Brazilian version of the Negative Acts Questionnaire-Revised, Campinas, SP, Brazil

Domains	R²	Q2	f²
Exclusion	0.821	0.437	0.388
Harassment	0.797	0.459	0.422
Quality/Overload	0.729	0.392	0.245
Undervaluation	0.607	0.351	0.192

**Figure 1.**
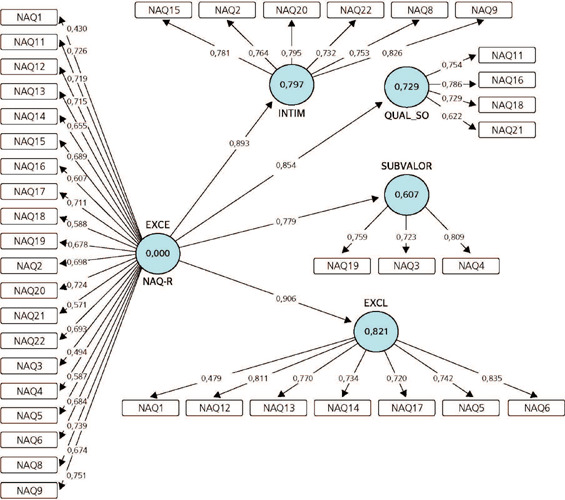
Final structural model of the Brazilian version of the Negative Acts Questionnaire-Revised (NAQ-R), Campinas, SP, Brazil. EXCL = Exclusion; HARAS = Harassment; NAQ = instrument variable; QUAL_OL = Quality/Overload; UNDRVL = Undervaluation.

Table 3 shows the high R² coefficient values, as proposed by Cohen: R² = 2% should be classified as small, R² = 13% as medium and R2 = 26% as large.^25^ The final step determined the path coefficient values, which are shown in Figure 1.

## DISCUSSION

This is the first Brazilian study to test the psychometric properties of an instrument that identifies bullying among nursing professionals in Brazil. The sample contained a disproportionate percentage of women, as has been reported in other studies of nursing professionals.

After the first round of tests, Item 7 "Having insulting or offensive remarks made about your person, attitudes, or your private life" and Item 10 "Hints or signals from others that you should quit your job" were removed to achieve acceptable values for discriminant validity. The validation study for the original NAQ-R^13^ mentioned the possibility of reducing the number of items without compromising the instrument's ability to measure bullying. This is due to cultural differences between countries, which affect behavior and organizational practices. These differences can affect the meaning of items in based on the selection and wording of the items.^13^

The reliability of the Brazilian version of the NAQ-R was verified, since the composite reliability values were > 0.80 for all domains, and the Cronbach's alpha values were > 0.70 in 3 of the 4 identified domains^26^. In further analysis, the R^2^ coefficient values were high,^26^ indicating that the domains were a good fit with the confirmatory factor model.

Table 3 shows that that the model has high Q2 values and that the Exclusion and Harassment domains were very important to the model. The importance of the Quality/Overload and Undervaluation domains were medium-high and medium, respectively. This analysis was based on f² values (Table 2), confirming, once again, the model's fit.

In the final step, the confirmatory model was analyzed by calculating the path coefficients. The high values indicated that all domains adhered to the confirmatory factor model and, thus, that the scale is capable of measuring bullying in the nursing work environment.

In Asian and European studies investigating the degree of evidence of the NAQ-R, modeling indicated 3 domains.^15,27,28,29^ This structure is similar to the original NAQ-R, which originated in Europe.^14^ However, the Portuguese version of the NAQ-R^17^ is an exception, since it classifies bullying into 4 domains, unlike versions of this instrument in other countries. Nevertheless, our discriminant validity results also resulted in 4 domains. This indicates that linguistic and cultural similarities can facilitate the adaptation of instruments for different populations.

Except for items 7 and 10, there were strong positive correlations between the items and domains. Item 7's exclusion might be due to the characteristics of bullying in professional environments, since performance and teamwork can be more important than the personal life of individual team members.

The exclusion of Item 10, which is about pressuring colleagues to quit, could have been related to the fact that approximately 30% of the sample has tenured positions through a civil service examination process. This creates a stronger bond between employees and the institution and reduced feelings of job insecurity, which has been associated with bullying in the literature.^30^ Furthermore, the involved health institution provides uncommon benefits regarding work hours and pay, even for staff hired through a regular employment contract (ie, with no job security).

As in the Portuguese version of the NAQ-R, confirmatory factor analysis supported the use of 4 domains and 20 valid, reliable items to measure bullying among nursing professionals. The adjusted model of the instrument is comprehensive and has potential for broad use in in Brazilian health services, allowing managers and health care professionals to more effectively recognize bullying behavior.

Although the sample included a significant number of professionals, some limitations should be considered. These stem from a lack of variables related to the institution's safety climate and the characteristics of the work units. Future research should use the instrument more comprehensively, considering a broader view of factors that could contribute to bullying behaviors among professionals.

## CONCLUSIONS

Confirmatory factor analysis demonstrated the validity of the Brazilian version of the NAQ-R. This instrument can be considered reliable and valid for assessing bullying among nursing professionals in Brazilian health services.

Through these results, we hope to provide managers of health care institutions with a reliable instrument that can identify acts of bullying among nursing professionals and enable prevention measures for this behavior, thus contributing to a positive organizational culture in health services.

## References

[1] Einarsen SV, Hoel H, Zapf D, Cooper CL (2020). Bullying and harassment in the workplace: theory, research and practice.

[2] Boudrias V, Trepanier SG, Salin D (2021). A systematic review of research on the longitudinal consequences of
workplace bullying and the mechanisms involved. Aggress Violent Behav.

[3] Conway PM, Erlangsen A, Grynderup MB, Clausen T, Rugulies R, Bjorner JB (2022). Workplace bullying and risk of suicide and suicide attempts: a
register-based prospective cohort study of 98 330 participants in
Denmark. Scand J Work Environ Health.

[4] Liu J, Gan Y, Jiang H, Li L, Dwyer R, Lu K (2019). Prevalence of workplace violence against healthcare workers: a systematic
review and meta-analysis. Occup Environ Med.

[5] AI Muharraq EH, Baker OG, Alallah SM (2022). The prevalence and the relationship of workplace bullying and nurses
turnover intentions: a cross sectional study. SAGE Open Nurs.

[6] Difazio RL, Vessey JA, Buchko OA, Chetverikov DV, Sarkisova VA, Serebrennikova NV (2019). The incidence and outcomes of nurse bullying in the Russian
Federation. Int Nurs Rev.

[7] Wunnenberg M (2020). Psychosocial bullying among nurse educators: exploring coping strategies
and intent to leave. J Nurs Scholarsh.

[8] Pogue CA, Li P, Swiger P, Gillespie G, Ivankova N, Patrician PA (2022). Associations among the nursing work environment, nurse-reported workplace
bullying, and patient outcomes. Nurs Forum.

[9] Karatuna I, Jönsson S, Muhonen T (2020). Workplace bullying in the nursing profession: a cross-cultural scoping
review. Int J Nurs Stud.

[10] Johnson AH, Benham-Hutchins M (2020). The influence of bullying on nursing practice errors: a systematic
review. AORN J.

[11] Anusiewicz CV, Ivankova NV, Swiger PA, Gillespie GL, Li P, Patrician PA (2020). How does workplace bullying influence nurses' abilities to provide patient
care? A nurse perspective. J Clin Nurs.

[12] Hawkins N, Jeong S, Smith T (2021). Negative workplace behavior and coping strategies among nurses: A
cross-sectional study. Nurs Health Sci.

[13] Einarsen S, Hoel H, Notelaers G (2009). Measuring exposure to bullying and harassment at work: Validity, factor structure
and psychometric properties of the Negative Acts Questionnaire-Revised.

[14] Einarsen S, Raknes Bl (1997). Harassment in the workplace and the victimization of men. Violence Vict.

[15] Makarem NN, Tavitian-Elmadjian LR, Brome D, Hamadeh GN, Einarsen S (2018). Assessment of workplace bullying: reliability and validity of an Arabic
version of the Negative Acts Questionnaire-Revised (NAQ-R). BMJ Open.

[16] Dujo-López V, González-Trijueque D, Grana-Gómez JL, Andreu-Rodríguez JM (2020). A psychometric study of a Spanish version of the Negative Acts
Questionnaire-Revised: confirmatory factor analysis. Front Psychol.

[17] Borges E, Ferreira T (2015). Bullying no trabalho: adaptação do Negative Acts
Questionnaire-Revised (NAQ-R) em enfermeiros. Rev Port Enferm Saude Mental.

[18] Kumari U, Muneer MZ, Murtaza MA, Abbas F, Sahito AM, Hassan Z (2023). Prevalence of workplace bullying among healthcare professionals in tertiary
care hospitals in Pakistan. Eval Health Prof.

[19] Tsai JC, Chang WP (2022). The mediating effect of job satisfaction on the relationship between
workplace bullying and organizational citizenship behavior in nurses. Work.

[20] Maciel RH, Gonçalves RC, Soboll LAP (2008). Violência psicológica e assédio moral no trabalho.

[21] Matos DAS, Rodrigues EC (2019). Análise fatorial.

[22] Ringle CM, Silva D, Bido DS (2014). Modelagem de equações estruturais com
utilização do Smartpls. REMark, Rev Bras Mark.

[23] Hair Jr JF, Hult GTM, Ringle CM, Sarstedt M (2014). A primer on partial least squares structural equation modeling (PLS-SEM).

[24] Forneil C, Larcker DF (1981). Evaluating structural equation models with unobservable variables and
measurement error. J Mark Res.

[25] Cohen J (1988). Statistical power analysis for the behavioral sciences.

[26] Hair Jr JF, Black WC, Babin BJ, Anderson RE (2019). Multivariate data analysis.

[27] Kakoulakis C, Galanakis M, Bakoula-Tzoumaka C, Darvyri P, Chrousos PG, Darviri C (2015). Validation of the Negative Acts Questionnaire (NAQ) in a sample of Greek
teachers. Psychology.

[28] Erwandi D, Kadir A, Lestari F (2021). Identification of workplace bullying: reliability and validity of
Indonesian version of the Negative Acts Questionnaire-Revised (NAQ-R). Int J Environ Res Public Health.

[29] Vukelic M, Čizmić S, Petrovic IB, Tenjović L, Giorgi G (2015). Psychometric properties of the Serbian version of the negative acts
questionnaire: revised. Psihologija.

[30] Bambi S, Guazzini A, De Felippis C, Lucchini A, Rasero L (2017). Preventing workplace incivility, lateral violence and bullying between
nurses: a narrative literature review. Acta Biomed.

